# Occurrence and dietary exposure of heavy metals in marketed vegetables and fruits of Shandong Province, China

**DOI:** 10.1002/fsn3.2485

**Published:** 2021-07-23

**Authors:** Tianran Zhang, Yuan Zhang, Wei Li, Lin Wang, Yanni Jiao, Yuxin Wang, Dafeng Jiang, Xibao Gao

**Affiliations:** ^1^ Department of Physical and Chemical Inspection School of Public Health Cheeloo College of Medicine Shandong University Jinan P. R. China; ^2^ Department of Physical and Chemical Testing Shandong Center for Food Safety Risk Assessment Shandong Center for Disease Control and Prevention Jinan P. R. China; ^3^ Department of Medical Examination Shandong Medical College Jinan P. R. China

**Keywords:** contamination levels, dietary exposure, food safety, health risk estimation, heavy metals, vegetables and fruits

## Abstract

The contamination of heavy metals in vegetables and fruits is a serious threat to food safety and human health. The present study was designed to investigate the occurrence and dietary exposure of lead (Pb), arsenic (As), cadmium (Cd), and mercury (Hg) in vegetables and fruits in Shandong Province, China. Results demonstrated that the mean level of total heavy metals was 30.25 µg/kg. The most frequently found heavy metal was Cd (69.2%) with a mean value of 11.54 µg/kg. The mean exposure values of Pb, As, Cd, and Hg in vegetables were 0.052, 0.045, 0.038, and 2.40 × 10^–3^ µg/kg bw/day, respectively. Moreover, the calculated hazard quotient (HQ) values of mean levels for these four heavy metals were all less than 1, indicating the health risk of heavy metal exposure caused by vegetable consumption was low. This study has displayed baseline information on heavy metal contamination in vegetables and fruits, which can provide useful data support for the formulation of relevant standards and government management.

## Graphical abstract

BLURB FOR ETOCThe study reported the occurrence and dietary exposure of lead (Pb), arsenic (As), cadmium (Cd), and mercury (Hg) in vegetables and fruits in Shandong Province, China. The calculated HQ values were less than 1, indicating the health risk of heavy metal exposure caused by vegetable consumption was low. The study displayed baseline information on heavy metal contamination of vegetables and fruits, which can provide data support for the formulation of relevant standards and government management.

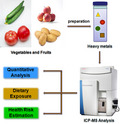

## 1 INTRODUCTION

Heavy metals usually refer to metals with a density greater than 5 g/cm^3^ (Nuapia et al., 2018; Rahman & Singh, 2019). Due to their accumulated toxicity, the contamination of heavy metals in food can interfere with the normal physiological function of the human body and then result in teratogenesis, carcinogenesis, and mutagenesis (Abdul et al., 2015; Chen et al., 2013; Järup, 2003). The use of metal‐based pesticides and fertilizers, irrigation with contaminated water, and contaminated soils can trigger the accumulation of heavy metals in plants. Then, they enter the food chain and have harmful effects on the human body (Chen et al., 2016; Hadayat et al., 2018). The elements of lead (Pb), arsenic (As), cadmium (Cd), and mercury (Hg) are all common heavy metals, which have been widely reported in water, air, soil, and other fields (Li et al., 2019; Suvarapu & Baek, 2017; Zhao et al., 2018). In particular, the contamination in food has received more and more attention, which has a threat to food safety and human health (Rai et al., 2019; Wang et al., 2020). Many organizations and countries have set maximum levels (MLs) of these heavy metals in food (Bi et al., 2018; Hadayat et al., 2018; Pan et al., 2016). For example, the European Union (EU) regulates an ML value of 500 µg/kg for Cd in leafy vegetables, and the ML value for As in tomatoes and cucumbers in Japan is 1,000 µg/kg (Hadayat et al., 2018). The Chinese government sets the MLs of Pb in meat and Hg in milk products, which are 500 µg/kg and 10 µg/kg, respectively (Tang et al., 2014).

Vegetables and fruits are essential components of the human diet because they contain key nutrients for human health, such as carbohydrates, proteins, vitamins, minerals, and fiber (Hu et al., 2013; Khillare et al., 2012). Both of them have a large number of consumers around the world. For instance, vegetable consumption in America grew by 19 percent from 1970 to 2005 and is expected to continue to grow through 2020 (Hadayat et al., 2018). China exports a lot of vegetables and fruits every year, and their domestic consumption is increasing gradually (Pan et al., 2016; Wang et al., 2015). More importantly, the safety of vegetables and fruits has attracted the attention of the governments and consumers worldwide. However, both vegetables and fruits are frequently contaminated by heavy metals (Osaili et al., 2016; Pan et al., 2016; Wang et al., 2015). The contamination could be occurred on the field and during processing, storage, and marketing. The contamination of Pb, As, Cd, and Hg in vegetables and fruits has been reported in published studies; however, only a few reports focused on the co‐occurrence of these heavy metals (Alagić et al., 2017; Guo et al., 2016; Jiang et al., 2020; Melai et al., 2018; Pajević et al., 2018; Wai et al., 2017). Therefore, it is necessary to simultaneously determine these heavy metals in vegetables and fruits and to estimate their health risks to humans.

Due to the low contamination levels of these heavy metals in vegetables and fruits, it is in great need of developing sensitive and accurate methods for simultaneous detection of different kinds of heavy metals. Conventional methods for the determination of heavy metals are atomic absorption spectrometry (Deng et al., 2020; Wang et al., 2015) and atomic fluorescence spectrometry (Bi et al., 2018; Pan et al., 2016). Inductively coupled plasma mass spectrometry (ICP‐MS) is an emerging method suitable for quantitative analysis of multicomponent elements. Compared with the above methods, ICP‐MS has the superiority of wide linear range, high sensitivity, high throughput, and strong antimatrix interference ability (Jiang, Huang, et al., 2020; Yin et al., 2020). It has been used in the applications of multicomponent metal elements in red wine, grain, meat, and eggs (de Freitas et al., 2013; Persson et al., 2009; Song et al., 2020; Vacchina et al., 2020). Therefore, ICP‐MS provides an ideal tool for the simultaneous detection of multicomponent heavy metals in food.

Shandong is a coastal province located in east China, which is one of China's major manufacturing and industrial provinces (Chai et al., 2017). Agriculture in Shandong Province is well developed; however, the information about heavy metal contamination in vegetables and fruits is limited. Therefore, the primary objective of the present study was to survey the contamination levels of Pb, As, Cd, and Hg in vegetables and fruits using ICP‐MS. The secondary objective of the present study was to estimate the dietary exposure of heavy metals in vegetables and fruits and compare them with health‐based guidance values. Finally, the health risks associated with heavy metal intake were assessed.

## 2 MATERIALS AND METHODS

### 2.1 Chemicals and reagents

The mixed standard stock solution (with concentrations of 20 *μ*g/mL for Pb, Cd, and As) was purchased from Inorganic Ventures (Virginia, USA). A concentration of 100 *μ*g/mL Hg standard stock solution [GBW(E)080124] was purchased from the National Institute of Metrology of China (Beijing, China). A mixed internal standard solution (Sc, Rh, In, and Bi) with the individual concentration of 1,000 *μ*g/mL was provided by the National Center for analysis and testing of nonferrous metals and electronic materials (Beijing, China). The instrument tuning solution (Li, Ba, Ce, In, and Co) with a concentration of 1 *μ*g/L was purchased from Inorganic Ventures (Virginia, USA). All reagents used in this study are of superior purity unless otherwise specified. Nitric acid was obtained from Merck (Darmstadt, Germany). A Milli‐Q purification water instrument (Millipore Co., USA) was utilized for the production of ultrapure water (resistivity 18.2 MΩ/cm).

### 2.2 Standard solutions and calibration curve

Firstly, the standard stock solution was diluted with nitric acid solution (2 + 98) appropriately to prepare a solution, in which the concentrations of Pb, Cd, and As were 20 µg/ml and that of Hg was 100 µg/ml. Then, a series of working solutions were prepared by properly diluting the above solution with nitric acid solution (2 + 98). The concentrations of Pb, Cd, and As were 0.0, 0.10, 0.50, 1.0, 10, 50, and 100 ng/ml, and the concentrations of Hg were 0.0, 0.05, 0.10, 0.50, 1.0, 1.5, and 2.0 ng/ml.

### 2.3 Sampling and sample preparation

In this study, 70 representative samples of stem vegetable (ginger, potato, yam, and onion), leafy vegetable (leek, scallion, celery, cabbage, romaine lettuce, Indian lettuce, and fennel seedling), and fruit vegetable (tomato, eggplant, cucumber, loofah, beans, and Cucurbita pepo), and 8 representative samples of fruit (strawberry, orange, apple, peach, watermelon, banana, and cherry) were obtained from local markets and retail stores in Shandong Province, China. Samples were taken between June and September in 2019. Each sample was stored at −20℃ before analysis and being homogenized. The inedible parts of some samples were discarded before pretreatment. It should also be noted that there is no further cooking procedure for the sample prior to analysis.

Briefly, about 0.5 g (accurate to 0.001 g) of the homogenized sample was weighed and transferred into a high‐pressure digestion tank. After that, 0.5 ml hydrogen peroxide and 5 ml nitric acid were added in the tank to digest the sample for 1 hr. The digestion solution was weighed to 25 g or volumized to 25 ml after being completely digested and well mixed for ICP‐MS analysis. At the same time, the reagent blank was prepared. The reagent blank, standard working solutions, and sample solutions were introduced into the ICP‐MS system for determination under the same instrumental conditions. Each sample was injected three times, and the measurement uncertainty was calculated using relative standard deviation (RSD).

### 2.4 ICP‐MS analysis and method validation

The concentrations of Pb, As, Cd, and Hg were measured by the Thermo iCAP Q ICP‐MS instrument (Thermo Fisher Scientific, Bremen, Germany). Qtegra^TM^ Intelligent Scientific Data Solution^TM^ software was used for data acquisition and analysis. The internal standard method was used for quantification. The developed assay was validated by linearity, the limit of detection (LOD), the limit of quantification (LOQ), accuracy, and precision. The LOQ was 3 times of the LOD. The recovery experiment was conducted to determine accuracy and intraday precision. The same procedure was repeated for three consecutive working days to achieve interday precision.

### 2.5 Dietary exposure estimation

EDI =C × dIR / BW.

EDI (µg/kg bw/day) is the estimated daily intake of each element; C (mg/kg) is the contamination level of target elements in vegetables; dIR (daily intake rate) (kg/day) is average daily consumption of vegetables; and BW (kg) is the mean value of body weight. The value of average dIR was obtained from the 2018 Shandong Province Health and Nutrition Survey, which was 0.2592 kg/day. The value of human body weight was set to be 60 kg.

### 2.6 Health risk estimation

Hazard quotient (HQ) is employed to evaluate the health risk associated with heavy metals caused by eating vegetables, which was the proportion of the EDI to the reference dose oral (RfD_O_) for metals (Luo et al., 2011). RfD_O_ represents the safe level of metal exposure by oral intake. HQ was calculated as:

HQ =EDI / RfD_O_.

The RfD_O_ values of Cd and As were 1.00 and 0.30 *μ*g kg^‐1^ day^‐1^, respectively, from the USEPA Integrated Risk Information System. According to EFSA and JECFA, the reference values of Pb and Hg were 1.50 *μ*g kg^‐1^ day^‐1^ and 0.14 *μ*g kg^‐1^ day^‐1^, respectively (Bi et al., 2018). The value of HQ >1 indicates potential risk, while the value <1 is safe.

## 3 RESULTS AND DISCUSSION

### 3.1 Method validation

Before analyzing the heavy metals in the real samples, the instrument method was validated using metal standard solutions. The validation data of the method are shown in Table 1. Standard curves of Pb, Cd, and As within 0.0–100 ng/ml and Hg within 0.0–2.0 ng/ml had excellent linearity, and the correlation coefficient (R^2^) was greater than 0.9985. The LODs and LOQs for Pb, As, Cd, and Hg were ranged from 0.1 to 3 *μ*g/kg and from 0.3 to 10 *μ*g/kg, respectively. Recovery experiments were conducted by spiking the samples with Pb, Cd, and As at levels of 50 *μ*g/kg and 500 *μ*g/kg (*n* = 6), with Hg standards at levels of 5 *μ*g/kg and 50 *μ*g/kg (*n* = 6), respectively. The recovery rate is 93.3%‐99.8% with high accuracy. The intraday precision and interday precision were represented by RSD. The intra‐RSD values were between 2.55% and 5.36%, while inter‐RSD values were between 2.16% and 6.61%. The results indicated that the current method displayed excellent precision and accuracy. Therefore, it was suitable for the determination of heavy metals in vegetables and fruits.

**TABLE 1:** Validation parameters of the proposed method

Heavy metals	r^2^	LOD (*μ*g/kg)	LOQ (*μ*g/kg)	Level 1 (50 *μ*g/kg)			Level 2 (500 *μ*g/kg)		
				Recovery (%)	Intra‐RSD (%)	Inter‐RSD (%)	Recovery (%)	Intra‐RSD (%)	Inter‐RSD (%)
Pb	0.9989	2	5	95.2	2.67	2.16	95.9	2.87	6.61
Cd	0.9992	0.1	0.3	99.1	5.36	5.16	99.8	3.72	4.46
Hg^a^	0.9985	1	3	97.4	3.63	5.15	94.6	2.66	4.97
As	0.9995	3	10	95.4	2.75	2.99	93.3	2.55	3.08

^a^ For Hg, the value of level 1 was 5 *μ*g/kg, and the value of level 2 was 50 *μ*g/kg.

### 3.2 Heavy metal concentrations in vegetables and fruits

The occurrence and contamination levels of Pb, As, Cd, and Hg in vegetables (*n* = 70) and fruits (*n* = 8) from Shandong Province are listed in Table 2. Briefly, the mean level of total heavy metals in samples was 30.25 µg/kg. The highest detection rate of heavy metals was Cd (69.2%), followed by Pb (32.1%), As (32.1%), and Hg (12.8%).

**TABLE 2:** Occurrence of heavy metals in vegetables and fruits in Shandong, China

Heavy metals	species	*n*	Mean (*µ*g/kg)	Range (*µ*g/kg)	Frequency/total (%)
Pb	Fruit vegetable	27	0	<LOQ^a^	25/78 (32.1%)
	Leaf vegetable	17	40.50	<LOQ−72.00	
	Stem vegetable	26	38.33	<LOQ−68.90	
	fruit	8	29.33	<LOQ−64.00	
Cd	Fruit vegetable	27	3.65	<LOQ−9.00	54/78 (69.2%)
	Leaf vegetable	17	8.03	<LOQ−30.00	
	Stem vegetable	26	18.34	<LOQ−49.94	
	fruit	8	0	<LOQ	
Hg	Fruit vegetable	27	9.11	<LOQ−9.11	10/78 (12.8%)
	Leaf vegetable	17	13.20	<LOQ−13.20	
	Stem vegetable	26	5.55	<LOQ−7.36	
	fruit	8	9.88	<LOQ−12.20	
As	Fruit vegetable	27	16.87	<LOQ−24.10	25/78 (32.1%)
	Leaf vegetable	17	40	<LOQ−138.00	
	Stem vegetable	26	18.54	<LOQ−29.20	
	fruit	8	0	<LOQ	

^a^ Lower than the reporting limit of quantification.

The range of Pb concentrations in vegetable and fruit samples was <LOQ‐72.00 µg/kg and <LOQ‐64.00 µg/kg, respectively. The mean concentrations in vegetables and fruits samples were 38.53 µg/kg and 29.33 µg/kg, respectively, which were below the National Standard of China (100 µg/kg). The order of mean contamination levels of Pb was leafy vegetable (40.50 µg/kg) > stem vegetable (38.33 µg/kg) > fruit vegetable (<LOQ). The highest Pb concentration in vegetable samples was found in romaine lettuce (72.00 µg/kg). In fruit samples, the highest value was found in peach (64.00 µg/kg), followed by apple (17.00 µg/kg) and banana (7.00 µg/kg), but it was not detected in other fruit samples. The results were compared with those in published studies. Hu et al., (2013) reported that the average concentrations of Pb from market vegetables of Hong Kong ranged from 50 to 170 µg/kg. Alagić et al., (2017) presented that the concentration of Pb in peach from Minićevo (Serbia) was 665 µg/kg DW (dry weight), which was much higher than the concentration of Pb in this study. The concentration of heavy metals in the eatable plant tissues of different kinds of vegetables was different, which might be caused by the different accumulation abilities of different elements in vegetables (Liu et al., 2013).

The range of Cd concentration in vegetable samples was <LOQ‐49.94 µg/kg. The mean concentration was 11.54 µg/kg, which was also below the National Standard of China (50 µg/kg). Cd was not detected in fruit samples. The order of mean contamination levels of Cd was stem vegetable (18.34 µg/kg) > leafy vegetable (8.03 µg/kg) > fruit vegetable (3.65 µg/kg). The potato had the highest concentration of Cd in vegetable samples (49.94 µg/kg). The results were similar to those of the previous study (Hadayat et al., 2018), which reported the level of metal accumulated in fruit vegetables was lower than that in leafy vegetables. Pajević et al., (2018) also confirmed that the concentration of Cd in fruit vegetables was lower than that in leaf and root vegetables. However, our result was different from that of Shaheenet al. (2016), which investigated Pb, Cd, As, Cu, Cr, Mn, Zn, and Ni concentrations in different vegetables (including brinjal, green chili, carrot, bean, potato, and tomato) grown in Bangladesh. They found that only Cd concentration in tomatoes (56 µg/kg) was above the permissible limit (50 µg/kg), which was much higher than the concentration of Cd in the present study. Pan et al., (2016) reported that the mean Cd concentration in apples was 17 µg/kg (fresh weight), which was also higher than that in this study. Wang et al., (2015) reported that the mean Cd concentration in apples was 4.90 µg/kg. Hu et al., (2013) demonstrated that 16% and 26% of vegetables in the Hong Kong market were contaminated with Cd and Pb. It could be explained that Cd is a toxic element, and plant tissues have different abilities to ingest and accumulate toxic elements (Li et al., 2016; Pajević et al., 2018; Singh et al., 2010).

The range of Hg concentrations in vegetable and fruit samples was <LOQ‐13.20 µg/kg and <LOQ‐12.20 µg/kg, respectively. The mean concentrations were 7.79 µg/kg and 9.88 µg/kg, respectively. In vegetable samples, the order of mean contamination levels of Hg was leafy vegetable (13.20 µg/kg) > fruit vegetable (9.11 µg/kg) > stem vegetable (5.55 µg/kg). The highest Hg concentration in vegetable samples was tested in Indian lettuce (13.20 µg/kg), which exceeded the maximum limits of 10 µg/kg set for Hg by the Chinese government. The results were similar to those of the previous study, which also reported that the concentration of Hg in leafy vegetables was higher than that in other vegetables (Zhong et al., 2018). Douay et al., (2013) showed that metals were more likely to accumulate in the foliar system than in the stored organs. Bi et al., (2018) reported that the mean Hg concentration in vegetables was 2 µg/kg (fresh weight), which was lower than that in this study. The concentration of heavy metals in leafy vegetables was higher than that in nonleafy vegetables, which may be because leafy vegetables have larger leaf areas and stronger surface adsorption capacity (Deng et al., 2020). Additionally, the highest Hg concentration in fruit samples was found in apple (12.20 µg/kg), followed by banana (10.30 µg/kg), peach (8.82 µg/kg), and cherry (8.21 µg/kg). It was not detected in other fruit samples. It should be pointed out that there is no maximum limit regulation of Hg in fruits in China.

The range of As concentration in vegetable samples was <LOQ‐138.00 µg/kg. The mean concentration was 29.23 µg/kg, which was below the National Standard of China (500 µg/kg). It was not detected in fruit samples. The order of mean contamination levels of As was leafy vegetable (40.00 µg/kg) > stem vegetable (18.54 µg/kg) > fruit vegetable (16.87 µg/kg). Romaine lettuce had the highest concentration of As in vegetable samples (138.00µg/kg). Hadayat et al., (2018) reported that tomato had the lowest As, while carrot had the highest As, which was different from the results of our study. Islam et al., (2015) and Rahman et al., (2013) reported that the mean concentrations of As in vegetables in Bangladesh were 200 and 50 µg/kg, respectively, which were both higher than those in this study.

### 3.3 Dietary exposure estimation

The quantification of common and high exposures to heavy metals was estimated using the mean estimated daily intake and the 97.5th percentile values, respectively (Pan et al., 2016). Table 3 demonstrates the estimated daily vegetable intake of Pb, As, Cd, and Hg for adults in Shandong Province. The highest mean estimated daily heavy metal intake in vegetables was Pb (0.052 µg/kg bw/day), followed sequentially by As (0.045 µg/kg bw/day), Cd (0.038 µg/kg bw/day), and Hg (2.40 × 10^–3^ µg/kg bw/day).

**TABLE 3:** Exposure assessment of heavy metals in vegetables from Shandong, China

Heavy metals	RfD_O_ ^a^ (*μ*g/kg/day)	EDI (*μ*g/kg bw/day)		Hazard quotient (HQ)	
		Mean	P97.5	Mean	P97.5
Pb	1.50	0.052	0.166	0.03	0.11
Cd	1.00	0.038	0.205	0.04	0.21
Hg	0.14	2.40 × 10^–3^	4.33 × 10^–2^	0.02	0.31
As	0.30	0.045	0.407	0.15	1.36

^a^ the reference dose oral.

The estimated dietary intakes of heavy metals were compared with the data acquired from the previous studies. Bi et al., (2018) showed that the estimated mean daily intakes of Pb, As, Cd, and Hg in leafy vegetables for suburban adults in Shanghai were 0.219 µg kg^‐1^ day^‐1^, 0.157 µg kg^‐1^ day^‐1^, 0.013 µg kg^‐1^ day^‐1^, and 0.198 µg kg^‐1^ day^‐1^, respectively. Most of them were higher than the exposure level of this study. Pan et al., (2016) showed that the mean daily intakes of As, Cd, Hg, and Pb by marketed vegetables in Zhejiang were 0.063, 0.083, 0.010, and 0.459 µg/kg bw/day, respectively, which were higher than the average value in this study. The mean exposure to Pb, As Cd, and Hg in this study was also lower than that reported by Bangladesh (Shaheen et al., 2016). Unlike previous studies (Hu et al., 2017), this study revealed that stem vegetables could accumulate more metals and could be with higher potential risk than fruit and leafy vegetables. Different types of vegetables might affect the value of EDI.

### 3.4 Health risk estimation

HQ was utilized to estimate the risk of food to human health (Luo et al., 2011; Nabulo et al., 2010). When HQ ≤1.0, the risk of vegetable metals was lower than that of RfD_O_, indicating low health risk (Ji et al., 2018). Table 3 displays that the mean HQ of As was <1, while the value of P97.5 was >1. This result indicated that common As exposure of vegetables posed low health risks, while high As exposure might have potential health risks. HQ mean value and P97.5 value of other metals were both <1, illustrating low health risk from vegetable consumption. The highest mean value of HQ for individual metals was As, followed sequentially by Cd, Pb, and Hg.

The estimated health risks were compared with the previous studies. Hu et al., (2017) reported that the highest value of HQ for vegetables along the Yellow Sea of China was As. Nabulo et al., (2010) reported that Cd and Pb had the highest HQ values among vegetables around Kampala, Uganda. Hu et al., (2013) reported that the highest potential health risk among vegetables in the Hong Kong market was Cd, and the second highest was Pb. Zhong et al., (2018) had calculated the total health risk index of people in Liaoning, Yunnan, Guizhou, Guangxi, Guangdong, Hunan, and Hubei provinces of China. The results demonstrated that the risks of Hg, Pb, and Cd in vegetables grown in these areas were high. In this study, the total metal levels were assumed as the real absorption values; therefore, the health risk of heavy metals may be overestimated. Regular monitoring of heavy metals and more specific exposure estimates in vegetables and fruits in the targeted area were recommended to ensure dietary safety.

## 4 CONCLUSIONS

In conclusion, the occurrence and contamination levels of heavy metals in marketed vegetables and fruits of Shandong Province in China were investigated in the present work. The contamination levels of Pb, As, Cd, and Hg in 70 vegetables and 8 fruits were quantified using ICP‐MS. Furthermore, the daily intake of these heavy metals was estimated and health risk assessment associated with dietary exposure was successfully evaluated. Results indicated that the heavy metal contamination of vegetables and fruits posed low health risks, but the current health risk assessment did not take account of other important sources of food contaminated with heavy metals. Hence, monitoring and risk assessment of heavy metals were still essential to maintain consumer health protection.

## CONFLICTS OF INTEREST

The authors declare that they have no known competing financial interests or personal relationships that could have appeared to influence the work reported in this paper.

## AUTHOR CONTRIBUTION

**Tianran Zhang:** Data curation (equal); Methodology (equal); Validation (equal); Writing‐original draft (equal). **Yuan Zhang:** Conceptualization (equal); Investigation (equal); Methodology (equal); Software (equal). **Wei Li:** Data curation (equal); Formal analysis (equal); Methodology (equal); Validation (equal); Visualization (equal). **Lin Wang:** Data curation (equal); Formal analysis (supporting); Investigation (equal); Validation (supporting). **Yanni Jiao:** Conceptualization (equal); Formal analysis (equal); Resources (equal); Software (equal). **Yuxin Wang:** Investigation (equal); Methodology (equal); Validation (equal); Writing‐original draft (equal). **Dafeng Jiang:** Conceptualization (equal); Funding acquisition (lead); Methodology (equal); Writing‐original draft (equal); Writing‐review & editing (equal). **Xibao Gao:** Funding acquisition (supporting); Methodology (equal); Writing‐review & editing (equal).

## ETHICAL STATEMENT

This study does not involve any human or animal testing.
